# Encounter Metrics and Exposure Notifcation

**DOI:** 10.6028/jres.126.003

**Published:** 2021-01-15

**Authors:** René Peralta, Angela Robinson

**Affiliations:** 1 National Institute of Standards and Technology, Gaithersburg, MD 20899, USA

**Keywords:** contact tracing, COVID-19, Diffie-Hellman, elliptic curves, encounter metrics, exposure notification, privacy-enhancing cryptography

## Abstract

We discuss the measurement of aggregate levels of encounters in a population, a concept we call encounter metrics. Encounter metrics are designed so that they can be deployed while preserving the privacy of individuals. To this end, encounters are labeled with a random number that cannot be linked to anything that is broadcast at the time of the encounter. Among the applications of encounter metrics is privacy-preserving exposure notifcation, a system that allows people to obtain a measure of their risk due to past encounters with people who have self-reported to be positive with severe acute respiratory syndrome coronavirus 2 (SARS-CoV-19), the cause of coronavirus disease 2019 (COVID-19). The precise engineering of a system for exposure notifcation should be targeted to particular environments. We outline a system for use in the context of a workplace such as the National Institute of Standards and Technology (NIST).

## 1 Introduction

Following the initial phase of the coronavirus disease 2019 (COVID-19) pandemic, there is a present need to normalize our societies as safely as possible. This requires careful consideration of how to repopulate office buildings, factory floors, public transport, and school and college campuses, among others. To this end, we propose the utility of *encounter metrics* — the measurement of aggregate levels of encounters in a population. The goal is to enable, in a privacy-preserving manner, measurements useful for making informed decisions about occupancy rates and mobility restrictions. For example, one might wish to evaluate the effect that a restriction of 20% vs. 40% occupancy in a building may have on the statistics of encounter metrics, or one might want to know whether the level of encounters in Building A is higher than in Building B and quantify the difference.

Encounter metrics could also prove useful for studying the effect of environmental modifications on levels of interaction within a population. Examples include restricting vs. enabling elevator access, putting an “occupied/free” sign on a bathroom so that only one person at a time uses it, or putting directional arrows in hallways as is done in some supermarket aisles. The knowledge gained through encounter metrics would be useful in designing our working environments so as to be more resilient to future pandemics and enable faster and more efficient responses when pandemics do occur.

Although our initial motivation was to address the COVID-19 pandemic, encounter metrics is neutral regarding the value or cost of encounters. The notion of encounter metrics is relatively new, and through this article we aim to highlight its value. Once the pandemic is over, these tools we propose could also be used as a way to promote social interaction in different environments. At a higher level, encounter metrics can be used to study the group dynamics of a set of autonomous agents in the field (e.g., cars, soccer players, sheep, troops).

As of this writing, there is an extensive literature on exposure notification as a response to the current pandemic. There are also hundreds of vendors of related technologies. We will not discuss all the distinct approaches and applications. Our goals are (1) to place the problem of exposure notification in the broader context of encounter metrics; (2) to call attention to significant privacy problems with most current implementations; and (3) to recommend a simple cryptographic solution to these problems.

## 2 An Encounter

When two parties *A* and *B* meet, a record of this encounter must be created. How this happens is a central design component of encounter metrics. To minimize communication requirements (the parties will typically carry resource-constrained devices), we assume that the encounter record can be created from broadcast information only. That is, there is no time or budget for two-way communication between *A* and *B*. Note that broadcasts could be heard by anyone, including possibly malicious parties.

The obvious way to label an encounter between two parties, *A* and *B*, is as an ordered pair (*ID_A_, ID_B_*), where *ID_X_* is a pseudonym for party *X* . We say this method is *based on device identities (IDs)*. Most current proposals for exposure notification are based on device IDs (see, for example, Refs. [1, 2]). They use random, short-lived pseudonyms as a way to promote privacy.

An alternative, which we call *encounter ID-based*, is to label each encounter with a single random number that is not linkable to the identities of *A* and *B* nor to whatever they broadcast. This is a privacy-by-design choice that will help avoid myriad privacy issues that arise in device ID-based methods.^1^1Defining privacy is very hard, and outside the scope of this note. We use the term privacy loss in a broad sense. This includes not only individuals’ (or their communities’) loss of anonymity, but also loss of autonomy, increased vulnerability to behavioral nudges [3, 4], loss of personal space, and more. This is of particular concern given that the protocols we propose may be run on personal telephones. “Smart” phones are currently allowed to operate as surveillance platforms. They record use and sensor data and transmit it to third parties without knowledge or effective consent of the user [5]. Any privacy loss incurred through use of an application (“app”) for encounter metrics, or exposure notification, could, in principle, be significantly magnified through correlation with the surveillance data already captured by the phone. Modern cryptography has tools for implementing encounter ID-based methods under the above restrictions. The Decisional Diffie-Hellman assumption over elliptic curves is as follows: Given a (suitably chosen) point *G* on an elliptic curve, and given *a · G* and *b · G*, where *a* and *b* are random integers, it is infeasible to distinguish *ab · G* from *r · G*, where *r* is a random integer. Under this assumption, which underlies much of modern public-key cryptography, we can securely define an encounter ID between parties *A* and *B* by

*ID_AB_* = (*ab*) *· G,* (1)

where

• *A* chooses *a* at random and broadcasts *a · G*;
• *B* chooses *b* at random and broadcasts *b · G*;
• both parties calculate (*ab*) *· G* using information they have (*a* or *b*) and the broadcast of the other party (*a · G* or *b · G*).

Note that only *A* and *B* can compute the encounter ID. A third party listening to the broadcasts cannot. In fact, even if the encounter ID is later made public, third parties are still not able to link that ID to *A* and *B*. This property is important in the exposure notification use case. The frequency with which devices change the random number they broadcast determines the *encounter duration*. For example, at the National Institute of Standards and Technology (NIST), we have been testing devices that broadcast random numbers that are updated every 60 s or so, while scanning every 30 s. When consecutive scans result in the same encounter ID, we heuristically record an encounter of duration 1 min.

We use Curve25519, an elliptic curve with 256-bit modulus (see [6] for the current NIST recommendations for elliptic curve cryptography). Quantum computers capable of threatening the Decisional Diffie-Hellman assumption may be available within a few decades. There exist post-quantum alternatives, which we do not discuss here. NIST is currently engaged in choosing post-quantum cryptographic protocols for standardization [7, 8]; the process will take a few years.

Dispensing with identities at the outset may seem to be a highly restrictive constraint. For example, will not parties need to know whether their present encounter is with a different party than their previous encounter? Perhaps surprisingly, identities are often not needed, depending on what population function needs to be computed and the assumptions we can make about the distribution of encounters. That topic is beyond the scope of this note; we refer the reader to Ref. [9].

## 3 Estimating Line-of-Sight Distance

At NIST, SaeWoo Nam and his collaborators have implemented the encounter ID approach on computer-attached “dongles” [10]. The initial scheme used any measurement of Bluetooth received signal strength indication (RSSI) value greater than -60 dBm in a 1 min time interval to indicate that there was an encounter that occurred within a distance of 2 m (about 6 feet) or less. One drawback of using only Bluetooth RSSI is that it does not account for the possibility of a wall between the dongles. Furthermore, RSSI can vary significantly depending on the position of the dongles and the surrounding environment. In the NIST team’s latest implementation, both RSSI strength and ultrasonic ranging are used to estimate line-of-sight distance between devices.

## 4 Agents at Fixed Locations

Besides autonomous mobile agents, a party to an encounter can be part of an infrastructure. For example, we might want to measure encounters with elevators, gates, stairways, supermarket aisles, hallways, buses, metro cars, etc. Note, however, that our definition of encounter ID does not reveal what types of agents were involved. Additional information will usually be required in the form of metadata. The privacy implications of this should be evaluated on a per-application basis.

## 5 Privacy Loss When Encounter IDs Are Made Public

When two agents *A* and *B* meet and obtain an encounter ID *X*, both agents automatically obtain an association between *X* and the other agent. Later, any public statement about *X* is linkable to identities by both *A* and *B* (although not by others). This can present a privacy problem. In the context of exposure notification (Sec. 9), we can mitigate the problem using secure multi-party computation so that each party learns only how many of their encounters involved infected persons. That is, the specific encounters that were with infected persons is not revealed.^2^2Of course, if all of a person’s encounters were with infected people then this does not help.

## 6 Statistical Inference

Different applications will have different definitions of an encounter between two parties. In Sec. 9 below, we define an encounter as “close for one minute,” with the definition of “close” being a system parameter. This allows the parameters to be set and updated on a per-application basis.

Applications will be able to detect encounters with varying levels of false positives and false negatives. These error rates will be technology dependent. However, encounter metrics does not need high accuracy. For statistical inference, it is often enough that the expected value of distance between devices is negatively correlated to RSSI (high RSSI values correspond to smaller distance). Encounter metrics is mostly about aggregate statistics, such as the number of encounters over time. If the number of encounters in a building is significantly higher today than yesterday, then something happened in the building or in the population, or both. Statistical inferences will be stronger when we can measure duration of encounters at higher resolution.

When using Bluetooth Low Energy (BLE), however, frequent scanning causes fast depletion of device batteries. The devices being tested at NIST scan approximately every 30 s. This allows them to set the unit of time for encounter duration to 1 min. The standard 3 volt battery used lasts several weeks.

## 7 Metadata

In foreseen applications, it would be useful to add metadata to the encounter ID. Time and location may be useful in various ways. For example, we may want to answer the question: “Is there too little distancing in the cafeteria at lunch time?” Group identifiers may also be useful for measuring encounters within subpopulations, for example, when attempting to answer questions like, “do younger schoolchildren have closer interactions than older schoolchildren?” or “do people in Building A have more frequent interactions than people in Building B?”, as well as quantifying those answers. Of course, metadata carry a risk of compromised privacy that must be assessed on a per-application basis.

## 8 Reporting Infrastructure for Encounter Metrics

A system for encounter metrics requires some way to compute population statistics. This could be posed as a problem from secure multi-party computation: The set of devices would like to compute a statistic of their private inputs (i.e. their encounters) without revealing the inputs themselves (see footnote 3 in Sec. 9). However, this may prove impractical.

Since we have defined encounter IDs to not contain personally identifiable data, it is likely that statistics can be computed by a central server without an unacceptable privacy loss. For encounter metrics, we will assume that each device anonymously communicates its encounter data to a central server. The server is trusted to honestly compute the target statistics. There are variations on this trust model that we will not discuss here.

The full engineering of an encounter metrics system and its reporting infrastructure will depend on the goal of the system and on the specifics of the target environment. In particular, it will depend on the trust assumptions that can be made about the participants. These assumptions, along with assumptions about the power of adversaries, form what is known as the security model. The security model for a secure campus such as NIST’s is very different from the model for an application at the general population level, so we cannot be specific. However, a general requirement is that the system needs to account for both privacy and security concerns. For example, it may be necessary to protect the system from false reporting by external attackers. The problem of preserving anonymity while showing that one is an authorized user can be solved using cryptographic group signatures [11].

## 9 Encounter Metrics and Exposure Notification for COVID-19

Evidence has grown that a significant spreading mechanism for COVID-19 is close encounters between infected individuals and the general population. Thus it would be useful to be able to measure how often this occurs, both at the population level and at the individual level. Automation of this process through the use of Bluetooth-enabled devices, either phones or dedicated dongles, is currently being pursued worldwide.

Encounter metrics inform of a “population risk” and of an “expected speed of transmission.” We would also like to enable a measure of individual risk. An individual is at higher risk of having contracted the disease if he or she has had encounters with one or more persons that have been diagnosed COVID-19 positive. The more such encounters, the higher the risk. Note that we are not measuring the duration of encounters explicitly. Instead, we set the duration of encounters at a low value (e.g. 1 min) and *count* the number of encounters. An encounter that lasts 10 min is recorded as 10 different encounters. This has some privacy advantages, simplifies implementation, and enables a natural measure of risk. Thus, we will use number of encounters with infected people as a proxy for the aggregate duration of such encounters. This means that the risk associated with many short encounters will be recorded as approximately the same as the risk of one long encounter [12].

Once an individual tests positive for the virus, it would be valuable for those with whom they came into contact in the recent past to be alerted. For privacy reasons, it might be desirable that alerts be anonymous to the extent possible. That is, an alert would be of the form “you have been exposed,” rather than of the form “Bob, whom you interacted with, tested positive.” Additionally, it would help if alerts came with a risk level, with higher risk being associated with more encounters with infected individuals. The working hypothesis is that virus transmission is positively correlated with the number of encounters.

Assuming an honest central server, one could enable exposure notification as follows:

• **Reporting:** An individual who is diagnosed can voluntarily and anonymously send all their encounter IDs to the central server.
• **Server storage:** The server maintains a running window (for example, of length 14 d) of reported encounter IDs.
• **Risk exposure notification:** Every day (or more often if real-time notification is advisable), each participating individual performs a two-party secure computation^3^3Multi-party secure computations are cryptographic protocols that allow a party to evaluate a function *f* (*·*) of private information distributed across a set of parties. In this case, there are two parties (the server and the user), each holding a set of encounter IDs. The target function *f* (*IDs_at server_, IDs_at user_*) is the cardinality of the intersection of the two sets. A secure two-party computation protocol for *f* could allow the user to obtain the cardinality of the intersection of the two sets and nothing more. The server would learn nothing with the server to obtain the *number* of encounter IDs that are both in their list and on the server’s list. The server does not learn this value.

The last step, risk exposure notification, can be performed via Epione, a very efficient protocol due to Trieu et al. [13].^4^4The Epione protocol actually reveals a little more than the cardinality of the intersection, as each party learns the cardinality of the other’s set. Note that only infected individuals report encounters. The total number of reported encounters, in principle known only to the server, is an important measure of population risk.

There are possible variations and enhancements of this protocol, some of which we briefly mention below. Also, there are a number of security issues that need to be addressed when not all players can be assumed to be honest. For example, it might be necessary to prevent individuals from falsely reporting encounters. If individuals must present a digital certificate from a health authority that attests to a positive diagnosis, then we must solve the problem of how to present this certificate to the server while preserving anonymity. This can be done efficiently using cryptographic tools (for example, via blind signatures [14] or zero-knowledge proofs [15]). We do not elaborate on these here.

Another problem when a player cannot be assumed to be honest is that the player can, in fact, determine which encounters in its list has been reported to the server. To determine if encounter with ID *X* has been reported, it can simply perform the Epione protocol with the set *D ∪{X}*, where *D* is a set of random numbers not corresponding to any encounters. The cardinality will be either 0 or 1. If it is 1, then *X* has been reported. This can be repeated multiple times to learn the status of all the device’s encounters, defeating the Epione protocol. A mitigation procedure would be to allow only one daily execution of the Epione protocol per device. Using cryptographic techniques, such as blind signatures, this can be achieved while maintaining anonymity. Several other cryptographic tools can be used to ensure players’ accountability without compromising the privacy of honest players.

In cryptography terminology, a semi-honest player is one who engages in the protocol honestly but then uses all information at their disposal to learn as much as they can. For example, the player in the previous paragraph is not semi-honest because they use dummy encounter IDs in the Epione protocol. There are environments where it is reasonable to assume that players are semi-honest, but there are many cases in which one cannot make this assumption. It is a considerable challenge to engineer an exposure notification system that mitigates all attacks and is efficient. The papers by Vaudenay [16, 17] contain a very good discussion of the security and privacy of current proposals for exposure notification.

### 9.1 The Epione Protocol

Suppose Alice and Bob have sets of numbers *{a*_1_, *. . ., a_n_}* and *{b*_1_, *. . ., b_m_}*, respectively. They are willing to collaborate in order to allow Alice to learn the cardinality of the intersection of their sets. Besides this cardinality, all they are willing to reveal to each other is *n* and *m*. The Epione approach uses elliptic curve cryptography roughly as follows (see Sec. 2):

1. Alice picks a random secret integer *r* and sends the values *{ra_i_ G |* 1 *≤ i ≤ n}* to Bob.
2. Bob picks a random secret integer *s* and sends, **in random order**, the values *{sra_i_ G |* 1 *≤ i ≤ n}* to Alice.
3. Alice divides by *r* to obtain the set *U* = *{sa_i_ G |* 1 *≤ i ≤ n}*, although she does not learn which value in the set corresponds to which *a_i_*.
4. Bob sends, **in random order**, *V* = *{sb_i_ G |* 1 *≤ i ≤ m}* to Alice.
5. Alice computes *card*(*U ∩V*).

The third step in the protocol requires Alice to “divide” an elliptic curve value by a constant she knows. This can be done efficiently provided the order of *G* in the elliptic curve is known.

Recently, Canetti *et al.* [18] proposed a protocol, called CleverParrot, that can be viewed as incorporating the Epione ideas directly into the encounter record. The protocol is roughly as follows (using elliptic curve notation):

1. Each player broadcasts *ts G*, where *t* is time and *s* is a secret known only to the player (the secret *s *remains constant for all broadcasts of this player).
2. Positively diagnosed players report the pair (*u, v*) = (*tr G, tsr G*) for each value *ts G* they heard while contagious, where *r* is a secret random number.
3. Each player can determine if reported encounter (*u, v*) is one of theirs by testing whether *us* = *v*, where *s* is the player’s secret.

### 9.2 Time and Enclosed Spaces

When two people, one after the other, ride an elevator, there may not be enough time between riders for the air in the elevator to be fully vented. If one places a stationary device in the elevator, then it can detect and record this situation. The device would need to be able to measure time elapsed.

Suppose that the stationary device *E* has an encounter *ID*_(_*_E,A_*_)_ and, *T* seconds later, it has another encounter with *ID*_(_*_E,B_*_)_. Then, if *T* is smaller than some threshold (e.g., 5 min), the device can report the pair (*ID*_(_*_E,A_*_)_, *ID*_(_*_E,B_*_)_) to the central server. If, for example, *ID*_(_*_E,A_*_)_ is later reported by *A* (i.e., *A* reports being diagnosed with the virus), then the server can add *ID*_(_*_E,B_*_)_ to the exposure notification list. Because encounter metrics uses encounter IDs rather than device IDs, the central server does not learn who was in the elevator. Note, however, that this requires the central server be told which encounter IDs involve the elevator. An alternative is for the elevator to report *ID*_(_*_E,B_*_)_ upon learning that *ID*_(_*_E,A_*_)_ was reported. However, this would require the elevator to learn which of its encounter IDs were reported, not just how many.

This should be considered, with appropriate parameters, for various enclosed places.

### 9.3 Privacy Concerns with Stationary Devices

There is a problem with the devices of Sec. 9.2, even if they are assumed to be semi-honest. Consider the example of a device in an elevator. When party *A* rides the elevator, an encounter ID *X*_(_*_A,E_*_)_ is associated with this event. Since the elevator is part of the building infrastructure, we should consider the possibility that it can communicate with other parts of the building infrastructure. In particular, if party *A* must swipe a credential to ride the elevator (this is the case in some elevators at NIST), then we should consider the possibility that the elevator associates a personal identity with encounter *X*_(_*_A,E_*_)_. Making this association is not a protocol violation. If, at a later time, party *A* gets a positive diagnosis for COVID-19, then it will report *X*_(_*_A,E_*_)_ to a central server. If preventing the central server from knowing the identity of party *A* is a privacy requirement, then one would have to prevent the elevator from sharing information with the server. This can be difficult to enforce and verify, particularly in situations where there is a difference of interests between management (whose interests lie in learning who is sick) and employees (who have a privacy interest).

### 9.4 The Problem With Systems Based on Device ID Rather than Encounter ID

Most of the exposure notification systems currently being deployed record device identifiers rather than encounter identifiers. To make it harder for device identifiers to be linked to people’s identities, they are typically short-lived random numbers. When an individual tests positive for COVID-19, all the identifiers used in the past 14 d are reported to a central server. These will include the identifiers that were broadcast as the user swiped a credential when entering a building or riding an elevator, or when in front of a security or traffic camera, or at a supermarket cashier or an automated teller machine, etc. As explained in Sec. 9.3, this carries significant risk of compromised security to the individual. It would be difficult to argue that the device IDs are anonymous. It should be considered that an authority may learn the personal identities associated with the device IDs. Perhaps more seriously, it should also be expected that external and undetectable parties can also make this association. The external party can avoid detection because it only has to monitor the broadcast of identified individuals. When encounter IDs are used instead, the attacker must both listen and broadcast (see Sec. 9.3), making them detectable. Because the use of encounter IDs instead of device IDs does not seem to incur significant costs, we do not recommend use of device IDs.

### 9.5 Quantifying Risk as a Function of the Duration of an Encounter

The infection probability is an exponential function (1 *− e^−λ x^*) of the duration *x* of the encounter [19]. The rate parameter *λ* of the distribution has not been established. Clearly, *λ* varies significantly with the type of encounter, but for the purposes of this paper, it can be thought of as an average across encounter types and subpopulations. Even when we are uncertain about the risk of encounters as a function of aggregate duration, the exponential model implies that if duration is doubled, then risk is approximately doubled. This should be very useful for informing non-pharmaceutical interventions [20].

## 10 Conclusion

Encounter metrics is a tool for gathering statistical data about interactions within a population of autonomous agents. The main unit of measurement is the encounter. During an encounter, two anonymous and highly constrained agents can each obtain an encounter ID. The encounter ID is unknowable even to parties monitoring all communication between the agents. Even if encounter IDs are made public, they cannot be linked to the agents.

Encounter metrics can be used to analyze the interactions within populations as we attempt to safely restart our societies during the COVID-19 pandemic of 2020-2021, and any future pandemic scenario. The aggregate encounter metric statistics will facilitate analysis of population interaction in buildings, comparisons across buildings or campuses, and data-driven adjustments in reopening processes. These measurements can also be valuable in designing our future working environments to be more resilient to spread of infectious diseases.

Another application of encounter metrics is exposure notification (also known as contact tracing). In this context, privacy is a critical requirement. It is important that the data gathered as part of exposure notification are minimal, subject to achieving the goals of the system. It is also important for the public to know exactly what is being captured, computed, and transmitted by devices used for exposure notification. From this perspective, it is useful to constrain the power of these devices as much as possible. Herein, we proposed a privacy-oriented exposure notification system based on encounter metrics and compared it to alternative systems that use random, short-lived device IDs instead of encounter IDs.

## References

[1] Rivest R, Weitzner D, Ivers L, Soibelman I, Zissman M (2020) PACT: Private automated contact tracing. Available at https://pact.mit.edu/

[2] Troncoso C, Payer M, Hubaux JP, Salathe´ M, Larus J, Bugnion E, Lueks W, Stadler T, Pyrgelis A, Antonioli D, Barman L, Chatel S, Paterson P, Čapkun S, Basin B, Beutel J, Jackson D, Roeschlin M, Leu P, Preneel B, Smart N, Abidin A, Gõrses S, Veale M, Cremers C, Backes M, Tippenhauer N, Binns R, Cattuto C, Barrat A, Fiore D, Barbosa M, Oliveira R, Pereira J (2020) Decentralized privacy-preserving proximity tracing. *arXiv preprint* arXiv:2005.12273. Available at https://arxiv.org/abs/2005.12273

[3] White MD (2010) Behavioral law and economics: The assault on consent, will, and dignity. New Essays in Philosophy, Politics & Economics: Integration and Common Research Projects, eds Gaus G, Favor C, Lamont J (Stanford University Press, Stanford, CA), pp 203–223.

[4] Hacker P (2016) Nudging and autonomy. A philosophical and legal appraisal. *Handbook of Research Methods in Consumer Law, Forthcoming*, eds Micklitz HW, Purnhagen K, Sibony A (Edward Elgar Publishing, Cheltenham, UK), pp 77–118. Available at https://ssrn.com/abstract=2779507.

[5] Zuboff S (2018) The Age of Surveillance Capitalism: The Fight for a Human Future at the New Frontier of Power (Public Affairs, New York) 1st Ed.

[6] Chen L, Moody D, Regenscheid A, Randall K (2019) Recommendations for discrete logarithm-based cryptography: Elliptic curve domain parameters (National Institute of Standards and Technology, Gaithersburg, MD), Draft NIST Special Publication (SP) 800-186.

[7] Chen L, Jordan S, Liu YK, Moody D, Peralta R, Perlner R, Smith-Tone D (2016) Report on post-quantum cryptography (National Institute of Standards and Technology, Gaithersburg, MD), NISTIR 8105.

[8] Alagic G, Alperin-Sheriff J, Apon D, Cooper D, Dang Q, Kelsey J, Liu YK, Miller C, Moody D, Peralta R, Perlner R, Robinson A, Smith-Tone D (2020) Status report on the second round of the NIST post-quantum cryptography Standardization Process (National Institute of Standards and Technology, Gaithersburg, MD), NISTIR 8309.

[9] Angluin D, Aspnes J, Diamadi Z, Fischer MJ, Peralta R (2006) Computation in networks of passively mobile finite-state sensors. Distributed Computing 18:235–253.

[10] NIST (2020) Exposure notification – protecting workplaces and vulnerable communities during a pandemic. National Institute of Standards and Technology, Gaithersburg, MD.

[11] Chaum D, Van Heyst E (1991) Group signatures. *EUROCRYPT’91: Proceedings of the 10th Annual International Conference on Theory and Application of Cryptographic Techniques* (Springer-Verlag, Berlin), pp 257–265. Available at https://dl.acm.org/doi/10.5555/1754868.1754897

[12] Haas C (2020) Quantitative microbial risk assessment. Available at https://chaasblog.wordpress.com/2020/05/18/its-the-dose-response-stupid/

[13] Trieu N, Shehata K, Saxena P, Shokri R, Song D (2020) Epione: Lightweight contact tracing with strong privacy. *arXiv preprint* arXiv:2004.13293. Available at https://arxiv.org/abs/2004.13293

[14] Chaum D Blind signatures for untraceable payments. Advances in Cryptology, eds Chaum D, Rivest RL, Sherman AT (Springer US, Boston, MA), pp 199–203. .

[15] Goldreich O, Micali S, Wigderson A (1986) Proofs that yield nothing but their validity and a methodology of cryptographic protocol design. 27th Annual Symposium on Foundations of Computer Science (sfcs 1986) (IEEE, Canada), pp 174–187. .

[16] Vaudenay S (2020) Centralized or decentralized? The contact tracing dilemma. *IACR Cryptol ePrint Arch* 2020:531. Available at https://eprint.iacr.org/2020/531.

[17] Vaudenay S (2020) Analysis of DP3T. *IACR Cryptol ePrint Arch* 2020:399. Available at https://eprint.iacr.org/2020/399.

[18] Canetti R, Kalai Y, Lysyanskaya A, Rivest R, Shamir A, Shen E, Trachtenberg A, Varia M, Weitzner DJ (2020) Privacy-preserving automated exposure notifcation. *IACR Cryptol ePrint Arch* 2020:863. Available at https://eprint.iacr.org/2020/863.

[19] Wilson AM, Aviles N, Petrie JI, Beamer PI, Szabo Z, Xie M, McIllece J, Chen Y, Son YJ, Halai S, White T, Ernst KC, Masel J (2020) Quantifying SARS-CoV-2 infection risk within the Google/Apple exposure notification framework to inform quarantine recommendations. medRxiv 10.1111/risa.13768 PMC8447042 34155669

[20] Grantz KH, Meredith HR, Cummings DA, Metcalf CJE, Grenfell BT, Giles JR, Mehta S, Solomon S, Labrique A, Kishore N, Buckee CO, Wesolowski A (2020) The use of mobile phone data to inform analysis of COVID-19 pandemic epidemiology. Nature Communications 11(4961). 10.1038/s41467-020-18190-5 PMC7528106 32999287

